# Overexpression of *NtGPX8a* Improved Cadmium Accumulation and Tolerance in Tobacco (*Nicotiana tabacum* L.)

**DOI:** 10.3390/genes15030366

**Published:** 2024-03-15

**Authors:** Xiang Peng, Tengfei Ma, Kejin Song, Xue Ji, Lien Xiang, Nan Chen, Ronglei Zu, Wenyi Xu, Shunqin Zhu, Wanhong Liu

**Affiliations:** 1 School of Chemistry and Chemical Engineering, Chongqing University of Science and Technology, Chongqing 401331, China; 2 College of Environmental Science & Engineering, China West Normal University, Nanchong 637009, China; 3 School of Life Science, Southwest University, Chongqing 400715, China

**Keywords:** cadmium, tobacco, NtGPX8a, oxidative stress, genome-wide analysis

## Abstract

Cadmium (Cd)-induced oxidative stress detrimentally affects hyperaccumulator growth, thereby diminishing the efficacy of phytoremediation technology aimed at Cd pollution abatement. In the domain of plant antioxidant mechanisms, the role of glutathione peroxidase (GPX) in conferring Cd tolerance to tobacco (*Nicotiana tabacum*) remained unclear. Our investigation employed genome-wide analysis to identify 14 *NtGPX* genes in tobacco, revealing their organization into seven subgroups characterized by analogous conserved domain patterns. Notably, qPCR analysis highlighted *NtGPX8a* as markedly responsive to Cd^2+^ stress. Subsequent exploration through yeast two-hybridization unveiled NtGPX8a’s utilization of thioredoxins AtTrxZ and AtTrxm2 as electron donors, and without interaction with AtTrx5. Introduction of *NtGPX8a* into *Escherichia coli* significantly ameliorated Cd-induced adverse effects on bacterial growth. Transgenic tobacco overexpressing *NtGPX8a* demonstrated significantly augmented activities of GPX, SOD, POD, and CAT under Cd^2+^ stress compared to the wild type (WT). Conversely, these transgenic plants exhibited markedly reduced levels of MDA, H_2_O_2_, and proline. Intriguingly, the expression of *NtGPX8a* in both *E. coli* and transgenic tobacco led to increased Cd accumulation, confirming its dual role in enhancing Cd tolerance and accumulation. Consequently, *NtGPX8a* emerges as a promising candidate gene for engineering transgenic hyperaccumulators endowed with robust tolerance for Cd-contaminated phytoremediation.

## 1. Introduction

The increasingly severe soil Cd pollution significantly impacts crop growth and yields, raising considerable agricultural safety concerns globally [1,2]. Excessive Cd accumulation in plants induces various forms of damage, with Cd-induced oxidative stress being the most prevalent [3,4]. Cd^2+^ does not directly induce the generation of reactive oxygen species (ROS) [5]. Previous studies have indicated that the mechanism by which Cd^2+^ triggers ROS production is its binding with Complex III in the electron transport chain (ETC), which impedes electron transfer [6]. Furthermore, NADPH oxidase-mediated “respiratory burst” serves as a primary source of Cd-induced ROS generation, where the nature and subcellular localization of NADPH oxidase (NOX) family members exhibit differential regulation in ROS metabolism [7,8]. Additionally, Cd, by displacing iron (Fe) or copper (Cu) ions in ferritin or metallothionein, induces hydroxyl radical production, considered an indirect pathway for ROS generation [9,10]. Once intracellular ROS concentrations surpass the defense threshold, cells enter an oxidative stress state, inducing cellular damage processes like lipid peroxidation, nucleic acid damage, and protein oxidation, posing a significant threat to plant cell survival [11]. Therefore, maintaining intracellular ROS homeostasis stands as a critical aspect in alleviating plant Cd^2+^ toxicity.

Phytoremediation is a technology that utilizes plants to remove, remediate, or mitigate pollutants in the environment. Compared to chemical or physical methods for treating heavy metal contamination, phytoremediation offers environmentally friendly, cost-effective, and efficient solutions [12,13]. The efficacy of this technology in addressing Cd contamination in the environment has been widely recognized [14]. Various factors influence the efficiency of phytoremediation, with the reduction in biomass of hyperaccumulators plants due to Cd toxicity being one of the key factors affecting remediation efficiency [15]. Therefore, seeking new hyperaccumulators or utilizing genetic engineering to enhance the heavy metal tolerance of hyperaccumulators is an effective strategy to address this issue.

Plants have evolved various strategies to alleviate oxidative stress induced by environmental stressors, where the antioxidant enzyme system acts as the primary defense [16]. The plant antioxidant enzyme system primarily comprises superoxide dismutase (SOD), catalase (CAT), peroxidase (POD), ascorbate peroxidase (APX), and glutathione peroxidase (GPX), forming a complex defense mechanism against oxidative damage caused by diverse stressors [17]. Numerous studies indicate that Cd^2+^ stress significantly induces the expression of plant antioxidant enzyme genes [18,19,20]. However, distinct heavy metals exhibit differential effects on the induction of antioxidant enzyme gene expression [21]. Unlike essential mineral nutrients like Cu, Fe, and Zn, Cd is not necessary for plant growth. By displacing metal ions at binding sites within proteins, Cd significantly diminishes the activity of antioxidant enzymes [22,23]. Consequently, the identification of Cd-insensitive antioxidant enzymes is crucial in enhancing plant resilience against Cd^2+^ toxicity.

GPX (EC 1.11.1.9) is a class of antioxidant stress enzymes within cells that utilize electron donors like glutathione to catalyze the reduction of H_2_O_2_ or organic hydroperoxides into water or non-toxic alcohols. Animal GPX plays a crucial role in regulating neural system development, suppressing iron-induced cell death in tumors, and scavenging reactive oxygen species induced by oxidative stress [24,25]. While plant GPX protein sequences exhibit high similarity to animal GPX, the active site in plant GPX proteins does not use selenocysteine. Moreover, these proteins utilize thioredoxin (Trx) instead of glutathione (GSH) as an electron donor [26]. Plant GPX family members are typically monomeric, and the expression levels of some members are strongly induced under Cd^2+^ stress [27]. Poplar PtGPX5 can chelate 32 Cd ions, suggesting its role not only in clearing intracellular peroxides induced by heavy metal stress but also in serving as a “Cd^2+^-sink” [28]. Unlike other antioxidant enzymes, the presence of Cd^2+^ does not reduce the enzyme activity of PgGPX in *Pennisetum glaucum* [29]. Therefore, plant GPX plays a significant role in scavenging Cd-induced ROS. However, the role of *N. tabacum* GPX under Cd^2+^ stress remains largely unknown.

The strategy of genome-wide analysis has successfully identified GPX family members in various plants, such as *Brassica napus* [30], *Cucumis sativus* [31], *Eutrema salsugineum* [32], and *Ammopiptanthus nanus* [33]. The expression levels of these genes are widely regulated in response to abiotic stress. *N. tabacum*, being globally one of the most extensively cultivated non-food economic crops, is considered a Cd hyperaccumulator [34]. Developing tobacco resources resistant to high levels of Cd holds the potential for applying them in remediating Cd-contaminated soil [35]. In this study, a genome-wide analysis strategy was employed to identify members of the *NtGPX* gene family in *N. tabacum*. The study utilized qPCR to analyze the Cd^2+^ stress-responsive *NtGPX* family genes and employed yeast two-hybrid experiments to screen for electron donors of NtGPX8a. Additionally, the biological function of NtGPX8a in resisting Cd^2+^ stress was confirmed in both *E. coli* and transgenic tobacco. These findings provide crucial insights into how NtGPX enhances plant resistance against Cd^2+^ stress by maintaining the homeostasis of ROS.

## 2. Materials and Methods

### 2.1. Genome-Wide Analysis and Identification of the NtGPX Family Genes

Based on previous research methodologies [21,36], we conducted a comprehensive genomic analysis of the tobacco *NtGPX* gene family. In summary, genomic sequences, coding sequences, and protein sequences of the tobacco variety TN90 were retrieved from the NCBI database (https://www.ncbi.nlm.nih.gov/ accessed on 31 January 2024). The candidate *NtGPX* (PF00255) genes were selected using the HMMER online tool (http://hmmer.org accessed on 31 January 2024) and validated using the Pfam (http://pfam.xfam.org/ accessed on 31 January 2024) and Smart databases (http://smart.embl.de/smart/ accessed on 31 January 2024). Ultimately, 14 *NtGPX* genes containing the conserved GSHPx domain were identified in tobacco. The relative molecular weights and theoretical isoelectric points (pI) of *NtGPX* family members were predicted using the ProtParam tool in ExPASy (http://www.expasy.org/ accessed on 31 January 2024). Subcellular localization of NtGPX was predicted using Plant-mPLoc (http://www.csbio.sjtu.edu.cn/bioinf/plant-multi/ accessed on 31 January 2024) and WoLF PSORT (https://wolfpsort.hgc.jp/ accessed on 31 January 2024). The Neighbor-Joining algorithm (Bootstrap = 1000) in MEGA11.0.11 software was employed to construct the phylogenetic tree of NtGPX, integrating GPX protein sequences from *Arabidopsis thaliana* [37], *Brassica Rapa* [30], *Oryza sativa* [38], and *Populus trichocarpa* [28]. The MEME tool was utilized to identify conserved motifs in NtGPX. GSDS was used to analyze the intron–exon patterns of the *NtGPX* gene family. The 2000 bp DNA sequence upstream of the *NtGPX* gene initiation codon was submitted to PlantCARE for *cis*-acting element analysis, and the data were visualized using corresponding tools in TBtools 1.108. The MUSELE tool in Jalview 2.11 software was employed for aligning and visualizing the comparison between NtGPX8a and AtGPX8 protein sequences. AlphaFold2 v1.5.5 online tool was used to predict the three-dimensional structure of NtGPX8a and AtGPX8 proteins. The predicted protein structures were compared using PyMOL 3.0 software, evaluating the confidence of the results based on the root-mean-square deviation (RMSD) values.

### 2.2. Plant Growth Conditions and Cd^2+^ Stress

Seeds of tobacco variety TN90 were sown in nutrient soil (peat: vermiculite: perlite = 3:6:1). The cultivation conditions included a temperature of 25 ± 2 °C, with a photoperiod of 16 h light and 8 h darkness. After two weeks, four tissues—roots, stems, young leaves, and mature leaves—of tobacco seedlings were collected for subsequent tissue-specific expression pattern analysis. To investigate the expression levels of the *NtGPX* gene family under Cd^2+^ stress, two-week-old tobacco seedlings were transplanted into 1/2 Hoagland nutrient solution and cultured for seven days. Subsequently, CdCl_2_ solution was added to the culture solution (final concentration 50 μM). Tobacco seedlings were harvested at 0, 12, 24, 48, and 72 h post-treatment for gene expression analysis of the shoots. This study employed leaf disk culture to analyze the primary physiological and biochemical parameters of transgenic and wild-type tobacco in response to Cd^2+^ stress. Briefly, healthy leaves from transgenic and wild-type tobacco were prepared into 2 cm leaf disks using a puncher. These disks were disinfected with 10% (*v*/*v*) NaClO solution for 5 min, then placed in Murashige and Skoog (MS) solid medium with or without CdCl_2_ (final concentration 50 μM). The culture conditions were maintained at 28 ± 2 °C with a photoperiod of 16 h light and 8 h darkness. The growth status was observed daily until photography.

### 2.3. RNA Extraction, Reverse Transcription, and qRT-PCR

Total RNA was extracted from plant materials using the RNAsimple Total RNA kit (No. DP419, Tiangen, Beijing, China). Subsequently, first-strand cDNA synthesis was carried out using the GoScriptTM Reverse Transcription System (No. A5003, Promega, Madison, WI, USA). The synthesized cDNA was utilized for subsequent qPCR analysis. The qPCR mixture was prepared according to the instructions provided with the NovoStart^®^ SYBR qPCR SuperMix Plus (No. E096-01A, Novoprotein, Suzhou, China). The qPCR was performed on the CFX96TM Real-Time PCR Detection System (Bio-Rad, Hercules, CA, USA). The PCR program was set as follows: 45 cycles, 1 min at 95 °C, followed by 15 s at 95 °C and 30 s at 60 °C. Primers for the *NtGPX* used in real-time PCR were designed and are listed in Table S3. Tobacco NtEF1α (accession number: AF120093) was employed as the reference gene, and the tobacco *NtGPX* gene family’s tissue expression pattern and Cd-induced expression pattern were calculated using the 2^−ΔΔCT^ method.

### 2.4. Heterologous Expression of NtGPX in E. coli and Cadmium Tolerance Assay

To verify the impact of NtGPX on Cd^2+^ tolerance, coding region fragments of *NtGPX4*, *NtGPX5b*, *NtGPX6a*, and *NtGPX8a* were cloned from tobacco variety TN90 (primer list in Table S3), and their sequenced lengths were determined as 513 bp, 513 bp, 699 bp, and 513 bp, respectively. After digestion with *Bam*H I and *Hind* III enzymes, the coding regions of these four genes were ligated into the protein expression vector pET28a. The resulting recombinant plasmids were then transformed into *E. coli* BL21 strain and sequenced for validation. To compare the effect of the four *NtGPX* genes on *E. coli* growth under cadmium stress, *E. coli* strains expressing these genes were cultured in LB liquid medium with or without Cd^2+^ (500 μM) (50 µg/mL Kan + 0.5 mM IPTG) at 28 °C and 180 rpm. The OD600 values were measured every two hours for a total of 4 times. The strain harboring the empty vector was used as a control. Additionally, to further understand the role of NtGPX8a in alleviating Cd^2+^ toxicity, the strain containing pET28::*NtGPX8a* was cultured on LB agar plates containing 500 μM CdCl_2_ at 37 °C for 24 h. The growth status was observed, and photographic records were taken.

### 2.5. Yeast Two-Hybrid

To ascertain potential electron donors for the NtGPX8a protein, following Zhang’s methodology [39], the yeast two-hybrid (Y2H) method was employed to validate interactions between NtGPX8a and three potential electron donor proteins sourced from *Arabidopsis*: AtTrx5 (AT1G45145), AtTrxz (AT3G06730), and AtTrxm2 (AT4G03520). Initially, bait vectors pGBKT7::*NtGPX8a* and prey vectors PGADT7::*AtTrxz*, PGADT::*AtTrx5*, PGADT7::*AtTm2* were constructed. These bait and prey vectors were co-transformed into Y2H Gold yeast strain using the LiTE-PEG method. Transformed colonies were selected on SD/-Leu-Trp plates, and the colonies were expanded in liquid SD/-Leu-Trp medium until reaching OD600 of 0.5–0.6. The culture was then diluted tenfold with sterile water, and subsequently, 6 µL of the diluted culture was spotted onto Double Dropout (DDO, SD/-Leu-Trp), Triple Dropout (TDO, SD/-Leu-Trp-His), and Quadruple Dropout (QDO, SD/-Leu-Trp-His-Ade) deficient media plates. The plates were incubated at 30 °C for 2–3 days, observing growth conditions and documenting results through photographic records.

### 2.6. Tobacco Genetic Transformation

The coding region of *NtPGX8a* was ligated into the pCXSN vector digested with *Xcm* I (NEB, Ipswich, MA, USA) to create the pCXSN::*NtGPX8a* recombinant plasmid. After sequencing validation, the recombinant plasmid was transformed into *Agrobacterium tumefaciens* strain GV3101. The leaf disk method was employed using Agrobacterium-mediated infiltration to genetically transform the TN90 tobacco variety [40]. Seedlings grown normally on plates containing 8 mg/L hygromycin were transplanted into the greenhouse. PCR was utilized to identify both the hygromycin resistance gene and the target gene, facilitating the selection of transgenic plants. Subsequently, total RNA was extracted from transgenic tobacco leaves and reverse-transcribed into cDNA. The expression levels of NtGPX8a in transgenic tobacco were analyzed using qPCR (primer details in Table S4). Leaf disks with a 2 cm diameter from lines L3 and L4, showing higher expression levels, were prepared for further research.

### 2.7. Determination of Cd^2+^ Content in E. coli and Transgenic Tobacco

To evaluate the chelating effect of NtGPX8a on cadmium, this study quantified the cadmium levels in *E. coli* expressing *NtGPX8a* and transgenic tobacco under cadmium stress. Bacterial cultures and dried tobacco leaves were collected, weighed, and subjected to dissolution by adding 5 mL of HNO_3_. The dissolution process was conducted on a hot plate at 100 °C until the solution clarified, then diluted to a final volume of 10 mL with distilled water. The cadmium content in the biological samples was determined using atomic absorption spectrophotometry at a wavelength of 228.8 nm.

### 2.8. Determination of Chlorophylls, Proline, TPC, and TFL Content

Following the Rainbow protocol published by Cristina López-Hidalgo et al. [41], the content of chlorophylls, proline, total phenolic compounds (TPC), and total flavonoid compounds (TFL) in both WT and transgenic tobacco leaf discs were determined. Briefly, plant materials were homogenized and centrifuged in 80% (*v*/*v*) ethanol, and the supernatant was collected. The absorbance at 470 nm, 649 nm, and 664 nm was measured using a microplate reader (Multiscan Go 1510, Thermo, Waltham, MA, USA), and the content of chlorophyll a, chlorophyll b, and carotenoids was calculated using the following Formulas (1)–(3).

C_*Chla*_ = 13.36 × *A*_664_ − 5.19 × *A*_649_ (1)

C_*Chlb*_ = 27.43 × *A*_649_ − 8.12 × *A*_664_ (2)

C_*Carotenoids*_ = (1000 × *A*_470_ − 2.13 × C_*Chla*_ − 97.63 × C_*Chlb*_)/290 (3)

(C: concentration, µg/mL; *A*: absorbance)

The proline content was determined using the ninhydrin colorimetric method. The supernatant was thoroughly mixed with 2% (*w*/*v*) ninhydrin reagent, and the absorbance at 520 nm was measured. Proline content was calculated based on the standard curve of L-proline + L-glutamine (1:1, *v*/*v*). The TPC content was measured using the Folin–Ciocalteu method. After mixing the supernatant with 10% (*v*/*v*) Folin–Ciocalteu reagent, absorbance at 720 nm was measured. TPC content was calculated based on the gallic acid standard curve. The TFL content in the samples was determined using the aluminum salt reagent complex colorimetric method. Samples were mixed with 10% (*w*/*v*) aluminum chloride, 1 M potassium acetate, and methanol. Absorbance was read at 415 nm, and TFL content was determined based on the quercetin standard curve.

### 2.9. Measurement of MDA and H_2_O_2_ Content

To evaluate the extent of oxidative damage in tobacco leaf discs under Cd^2+^ stress conditions, the levels of MDA and H_2_O_2_ in each of the tobacco plants were measured using Lipid Peroxidation (MDA) Assay Kit (No.0131S, Beyotime, Haimen, China) and Hydrogen Peroxide Assay Kit (No. A064-1-1, NJJCBIO, Nanjing, China), respectively. All experimental procedures were carried out following the instructions provided in the respective assay kits. The detection wavelengths for MDA and H_2_O_2_ were 535 nm and 560 nm, respectively.

### 2.10. Measurement of GPX, SOD, CAT, POD, and GR Enzyme Activities

The GPX activity in tobacco leaf discs was determined using a Total Glutathione Peroxidase Assay Kit (No. S0059S, Beyotime, Haimen, China), while the SOD activity was assessed using a Total SOD Assay Kit (No. S0101S, Beyotime, Haimen, China). The enzyme activities of POD, CAT, and GR were measured according to the instructions provided in the Biochemical Assay Kits (No. A084-3-1, No. A007-1-1, No. A062-1-1, NJJCBIO, Nanjing, China). The absorbance of GPX (λ = 340 nm), SOD (λ = 550 nm), POD (λ = 420 nm), CAT (λ = 405 nm), and GR (λ = 340 nm) reaction mixtures was recorded using an enzyme analyzer, and enzyme activities were calculated using the formulas provided in the kit instructions.

### 2.11. Statistical Analysis

Unless otherwise specified, a minimum of three biological replicates were used for data computation, and the results are presented as mean ± SDs. The statistical analysis of all experimental data was performed utilizing either a *T*-test or one-way ANOVA. Significance among groups was determined using Dunnett’s multiple range test in GraphPad Prism 8.0.2 (GraphPad Software, San Diego, CA, USA).

## 3. Results

### 3.1. Genome-Wide Identification and Characterization of NtGPX Gene Family

**Figure 1:**
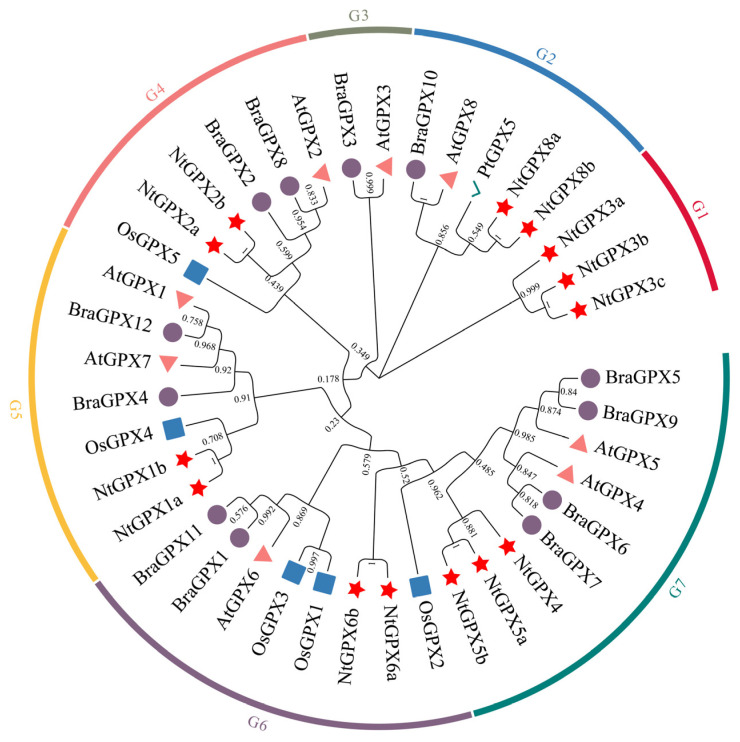
Phylogenetic analysis of GPX proteins from *N. tabacum*, *A. thaliana*, rice, *B*. *rapa*, and *P*. *trichocarpa*. NtGPX is represented by a red pentagram, AtGPX by a green circle, OsGPX by a yellow rectangle, BraGPX by a blue triangle, and a gray checkmark represents the PtGPX5 that possesses cadmium chelation capability.

Based on the genome-wide analysis of the TN90 tobacco variety, we identified 14 members within the *NtGPX* gene family. To understand the evolutionary relationships of the *NtGPX* gene family, we constructed a phylogenetic tree comprising 40 plant GPX proteins from *N. tabacum*, *A. thaliana*, rice, *B. rapa*, and *P. trichocarpa* (Figure 1). Concurrently, we named the *NtGPX* family members based on their closest evolutionary relationships with *A. thaliana* genes. The phylogenetic analysis revealed the clustering of 40 GPX proteins into seven distinct clades (designated as G1–G7). Notably, unlike *A. thaliana*, the tobacco *NtGPX* family does not include members of the III subfamily. Among these, the NtGPX8 subfamily clustered with PtGPX5, a known Cd^2+^ chelating protein, indicating that members of the NtGPX8 subfamily may possess biological functions akin to PtGPX5′s Cd^2+^ chelation abilities.

In this study, we conducted a comprehensive bioinformatic analysis of the *NtGPX* gene family. The 14 genes within the *NtGPX* family encode proteins ranging from 157 to 294 amino acid residues, with predicted pI values ranging between 4.91 and 9.39. Additionally, subcellular localization predictions suggest that *NtGPX* family members might be located in the cytoplasm, chloroplasts, mitochondria, and nuclei, hinting at potential functional variations among these members (Table S1). Multiple sequence alignments revealed three highly conserved structural domains (G1–G3) within the *NtGPX* family, where the conserved Cys within the G1 domain represents a key residue for GPX activity (Figure S1). Interestingly, three members of the NtGPX3 subfamily exhibit longer differing sequences between the G2 and G3 domains. Coupled with the results from the phylogenetic tree, these findings suggest that members of the NtGPX3 subfamily might have distinct origins from other NtGPX members. Analysis of conserved domains and gene structures showed that all NtGPXs contain motif 3, with substantial differences in gene structure and motif composition among different subgroups of NtGPX (Figure S2). Moreover, we predicted cis-regulatory elements in the promoter regions of the *NtGPX* family genes involved in hormone response, stress response, and developmental regulation (Table S2). Notably, the promoter region of NtGPX6a contains the highest number of regulatory elements, including eight involved in MeJA responsiveness (Figure S3).

### 3.2. Tissue Expression Patterns and Cadmium-Induced Expression Patterns of NtGPX Family Genes

**Figure 2:**
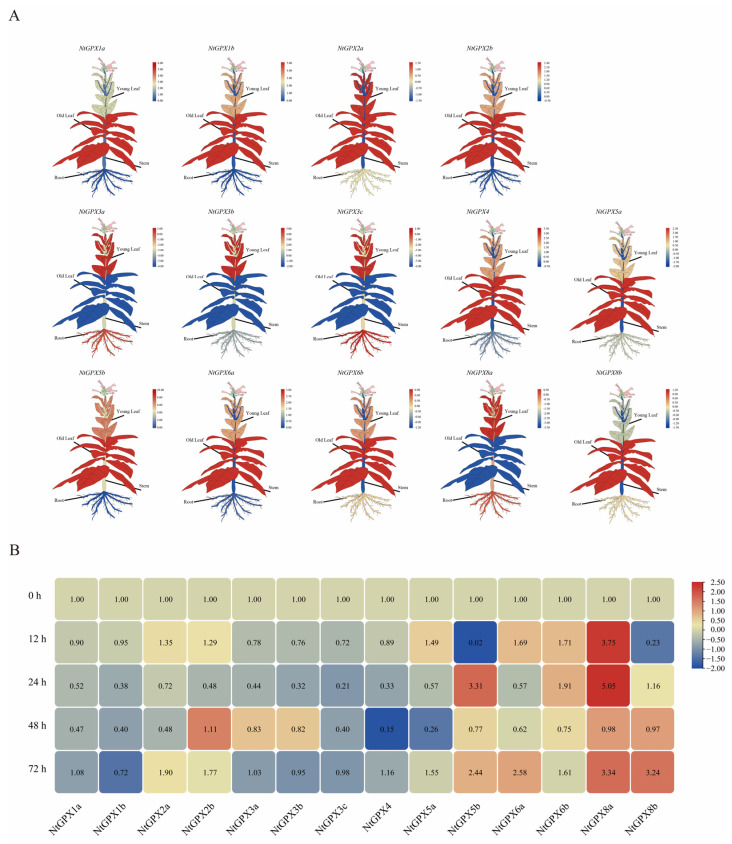
Analysis of tissue expression patterns and cadmium (Cd)-induced expression patterns of 14 members within the *NtGPX* gene family. (**A**) The relative expression levels of *NtGPX* genes in different tobacco tissues. (**B**) Expression of the tobacco *NtGPX* gene family under cadmium stress. The expression data were obtained from the real-time RT-PCR (qPCR) analysis and are shown as log2 values calculated as averages. The expression level of *NtGPX* in the root is defined as 1 (log2 = 0). High expression levels are shown in red, and lower expression levels are shown in blue.

In this study, we employed qPCR to analyze the tissue expression patterns and cadmium (Cd)-induced expression patterns of the 14 members within the *NtGPX* gene family. Tissue expression patterns were examined in tobacco plants’ roots, stems, young leaves, and mature leaves under normal growth conditions (Figure 2A). qPCR results revealed six genes, *NtGPX1*, *NtGPX2*, *NtGPX4*, *NtGPX5*, *NtGPX6*, and *NtGPX8b*, exhibiting higher expression levels in mature leaves. In contrast, the two genes with higher expression in young leaves were *NtGPX3* and *NtGPX8a*. Notably, the genes showing elevated expression in roots included *NtGPX3a*, *NtGPX3c*, and *NtGPX8a*, whereas *NtGPX8a*, *NtGPX3a*, *NtGPX3b*, and *NtGPX3c* displayed higher expression in stems. Overall, the tissue expression pattern of *NtGPX8a* resembled that of the *NtGPX3* subfamily, while other *NtGPX* family genes showed distinct tissue expression patterns.

To decipher the impact of Cd^2+^ stress on the expression levels of *NtGPX* family genes, we assessed the Cd-induced expression profile in tobacco leaves treated with 50 μM CdCl_2_. Results revealed that the expression levels of certain *NtGPX* family genes were affected by Cd^2+^ stress (Figure 2B). During the early stages of Cd^2+^ stress (≤24 h), genes within the *NtGPX1*, *NtGPX2*, *NtGPX3*, and *NtGPX4* subfamilies exhibited significant downregulation in expression. For instance, the expression level of *NtGPX3c* was only 0.21 times that of the control after 24 h of Cd^2+^ treatment. However, during the later stages of Cd^2+^ stress (48–72 h), the expression levels of these genes reverted to normal levels. Conversely, most members of the *NtGPX5*, *NtGPX6*, and *NtGPX8* subfamilies showed upregulation in their expression levels during the early stages of Cd^2+^ stress, maintaining relatively higher expression levels even in the later stages of stress. Notably, *NtGPX8a* exhibited a substantial increase in expression levels compared to the control at 12 h, 24 h, and 72 h of Cd^2+^ treatment, showing 3.75, 5.05 and 3.34 times higher expression, respectively. These findings suggest that NtGPX8a might play a significant role in tobacco’s response to Cd^2+^ stress, exhibiting the strongest inducible response among the *NtGPX* family genes.

### 3.3. Heterologous Expression of NtGPX8a Confers Cadmium Tolerance in E. coli and Enhances Cadmium Accumulation

**Figure 3:**
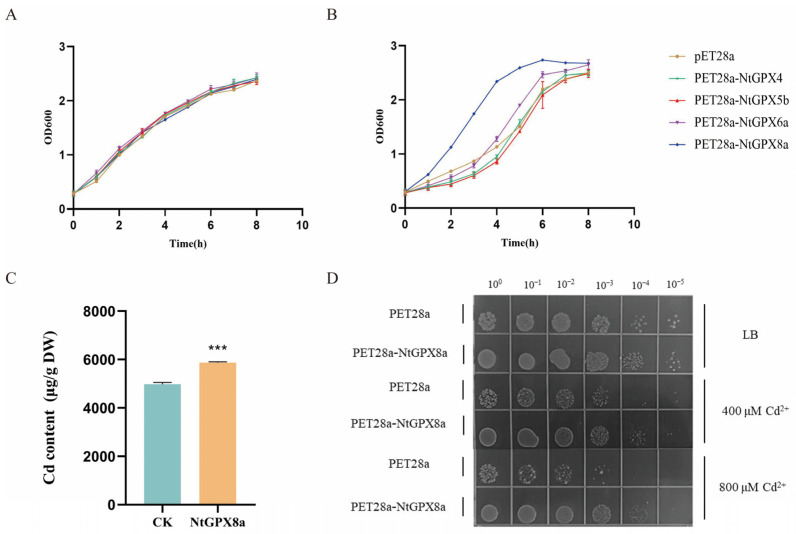
Analysis of stress resistance of heterologous expression of *NtGPX8a* in *E. coli*. (**A**) The growth curves of five *E. coli* strains, expressing heterologous genes, with an initial OD600 = 0.6 were plotted under normal conditions. The cultures were monitored at intervals of 0 h, 2 h, 4 h, 6 h, and 8 h, respectively. (**B**) The growth curves of five *E. coli* strains, expressing heterologous genes, with an initial OD600 = 0.6 were plotted under 500 μM CdCl_2_ treatment. The cultures were monitored at intervals of 0 h, 2 h, 4 h, 6 h, and 8 h, respectively. (**C**) Cadmium enrichment of *E. coli* heterologously expressing *NtGPX8a* and empty vector (CK) under 500 μM CdCl_2_ treatment. (**D**) Growth of *E. coli* strains heterologously expressing *NtGPX8a* and empty plasmid in gradient dilutions of culture medium containing different concentrations of Cd. Asterisks in the figure indicate the statistical significance of the difference calculated using the unpaired sample *T*-test, *** means *p* < 0.001, *n* = 3.

To elucidate the impact of NtGPX on mitigating Cd^2+^ toxicity, this study constructed prokaryotic expression vectors for *NtGPX4*, *NtGPX5b*, *NtGPX6a,* and *NtGPX8a*, individually, and introduced them into the *E. coli* BL21 strain. Under LB liquid culture conditions, the growth curves of the five *E. coli* strains harboring either the recombinant plasmid or the empty vector exhibited similar trends (Figure 3A). However, in LB liquid medium supplemented with 500 μM CdCl_2_, the OD600 values of the strain expressing *NtGPX8a* were significantly higher than those of the other four groups (Figure 3B). Intriguingly, the OD600 values of *E. coli* expressing *NtGPX8a* in Cd-containing LB medium were even higher than those in the absence of Cd (Figure 3A,B). For instance, at 4 h of incubation, the OD600 for *NtGPX8a*-expressing bacteria was OD600 = 2.34 in Cd-containing medium compared to OD600 = 1.65 in Cd-free medium. This implies that *NtGPX8a* not only effectively mitigates the toxicity of Cd^2+^ to bacteria but may also promote bacterial growth. To further confirm the role of *NtGPX8a* in alleviating Cd^2+^ toxicity, we conducted Cd^2+^ tolerance assays using solid LB medium with or without CdCl_2_. In Cd-free LB medium, the growth of *E. coli* harboring pET28a::*NtGPX8a* recombinant plasmids and those with pET28a vectors was nearly identical (Figure 3D). However, in media containing 400 μM or 800 μM CdCl_2_, the growth of the strain expressing *NtGPX8a* significantly outperformed the control.

A plausible reason for the favorable growth of *NtGPX8a*-expressing strains under Cd^2+^ stress could be the relatively lower intracellular Cd levels. To validate this hypothesis, atomic absorption spectroscopy was used to measure Cd content in *E. coli* strains expressing *NtGPX8a* or the empty vector under 500 μM CdCl_2_ treatment. Surprisingly, the heterologous expression of pET28a::*NtGPX8a* resulted in an intracellular Cd content of 5872.06 μg/g, 1.18 times higher than that of the control group (Figure 3C). These findings indicate that the heterologous expression of *NtGPX8a* not only effectively mitigates Cd^2+^ toxicity in *E. coli* but also enhances Cd accumulation within the bacterial cells.

### 3.4. Structure of NtGPX8a and Selection of Electron Donors

**Figure 4:**
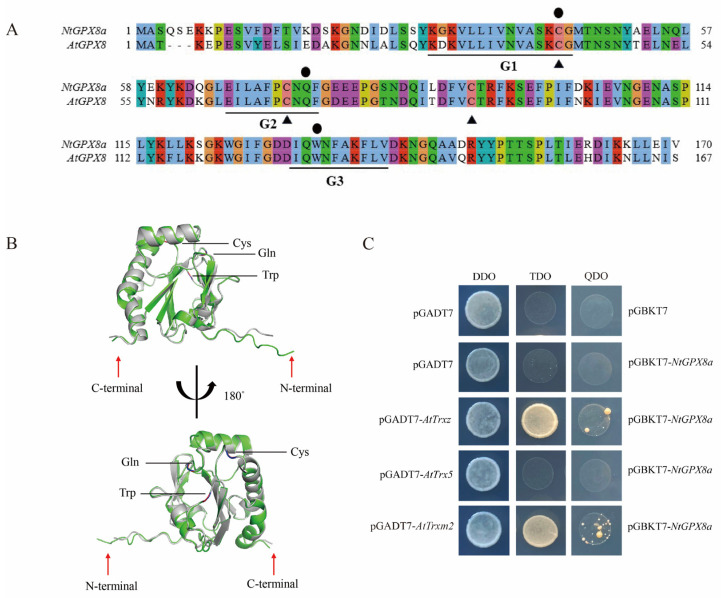
Analysis of NtGPX8a structure and electron donor. (**A**) Sequence alignment of the NtGPX8a and AtGPX8. Triangles denote three conserved Cys residues, while circles indicate the three amino acids (Cys, Gln, Trp) forming the catalytic triad. G1, G2, and G3 represent highly conserved characteristic domains. (**B**) Three-dimensional structural comparison between NtGPX8a and AtGPX8. NtGPX8 is depicted in green, AtGPX8a is shown in grew. (**C**) Interaction analysis of tobacco NtGPX8a with AtTrxz, AtTrx5, and AtTm2.

Protein functionality is often dictated by its three-dimensional spatial structure. Comparative sequence alignment between NtGPX8a and AtGPX8 revealed high consistency, with only one amino acid residue differing within the three conserved motifs. Furthermore, three crucial amino acid residues associated with GPX activity (Cys44, Gln 75, Trp133) and three conserved Cys residues were highly consistent between NtGPX8a and AtGPX8 (Figure 4A). We utilized PDB100 as templates to predict the 3D protein structures of AtGPX8 and NtGPX8a separately in AlphaFold2, resulting in five predicted protein models. Subsequently, the protein structure model with the highest Predicted Inter-residue Distance Distribution Transformation (pIDDT) score was selected for visualization. The result demonstrated a remarkably high structural similarity between the protein three-dimensional structures of NtGPX8a and AtGPX8 (RMSD = 0.301 Å) (Figure 4B).

Based on these predictive results, three thioredoxins (Trxs) from *A. thaliana*, including AtTrx5 (h-type), AtTrxZ (p-type), and AtTrxm2 (m-type), were selected to investigate the potential electron donors for NtGPX8a. Yeast two-hybrid results indicated that yeast strains co-expressing pGBKT7::*NtGPX8a* and GADT7::AtTrxz or pGBKT7::*NtGPX8a* and PGADT7::*AtTrxm2* grew on TDO and QDO plates, whereas yeast strains co-expressing pGBKT7::*NtGPX8a* and PGADT7::*AtTrx5* did not show growth (Figure 4C). This outcome suggests that NtGPX8a selectively interacts with specific Trxs as electron donors.

### 3.5. Overexpression of NtGPX8a Alleviates Cd-Induced Decrease in Photosynthetic Pigments and Enhances Cd Accumulation

**Figure 5:**
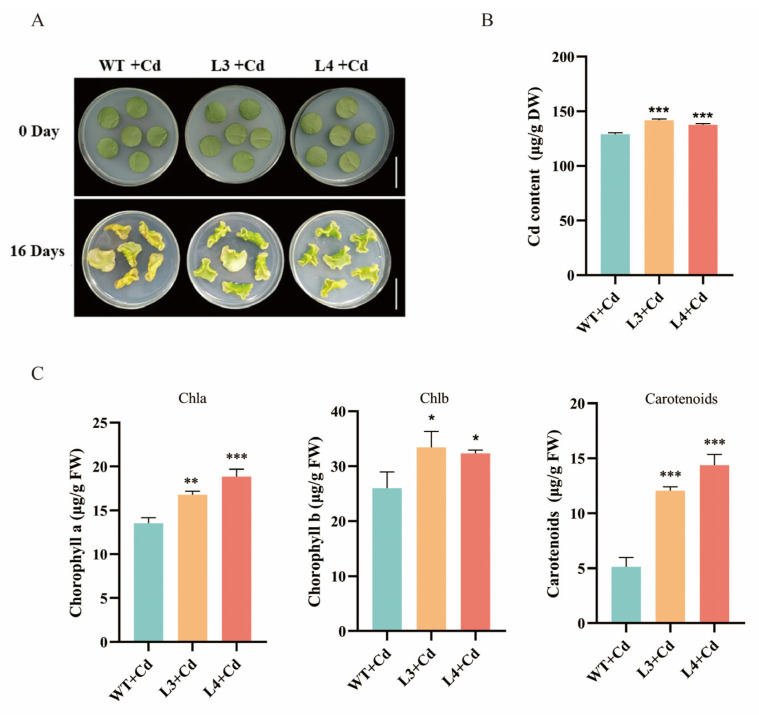
Analysis of tobacco leaf disk phenotypes and photosynthetic pigment content under cadmium stress. (**A**) Assessment of leaf disc growth status under 50 μM Cd^2+^ treatment for both 0 and 16 days, with a reference scale bar of 3 cm. (**B**) The Cd content in tobacco leaf disks after exposure to 50 μM Cd for a duration of 16 days. (**C**) Quantification of chlorophyll a, chlorophyll b, and carotenoid contents in tobacco leaf discs exposed to Cd^2+^ stress. All statistical analyses were performed by one-way ANOVA, where * represents *p* < 0.05, ** represents *p* < 0.01, *** represents *p* < 0.001, *n* = 3.

To elucidate the role of *NtGPX8a* in tobacco’s response to Cd^2+^ stress, we employed agrobacterium-mediated genetic transformation to obtain four independent transgenic tobacco lines labeled as L1 to L4. qPCR results indicated that the expression levels of *NtGPX8a* in transgenic tobacco lines L1 to L4 were 30.38, 34.30, 38.29, and 72.80 times higher compared to the wild type (WT) (Figure S4). Among these lines, L3 and L4, exhibiting the highest *NtGPX8a* expression levels, were selected for subsequent experiments. Leaf discs from transgenic lines L3, L4, and WT were cultured on MS solid medium containing 50 μM CdCl_2_ for 16 days. Results revealed that wild-type leaf discs exhibited curling and chlorosis, while leaf discs from transgenic lines L3 and L4 displayed significantly better growth than the wild type (Figure 5A). Furthermore, we assessed the contents of three photosynthetic pigments. Chlorophyll a content in L3 and L4 leaf discs was 1.24 and 1.39 times higher than that in WT, respectively. Similarly, chlorophyll b content was 2.01 and 1.95 times higher, and carotenoid content was 2.35 and 2.81 times higher in L3 and L4, respectively, compared to WT (Figure 5C).

Simultaneously, flame atomic absorption spectroscopy was used to measure Cd content in transgenic tobacco lines L3, L4, and WT under Cd^2+^ stress. The Cd content in WT, L3, and L4 was measured as 128.90 (μg/g DW), 141.90 (μg/g DW), and 137.60 (μg/g DW), respectively (Figure 5B). This aligns with the earlier findings from *E. coli*, where overexpression of NtGPX8a led to increased Cd accumulation. In summary, the overexpression of *NtGPX8a* in tobacco not only effectively alleviated Cd-induced chlorosis but also facilitated Cd accumulation in the plant.

### 3.6. Overexpression of NtGPX8a Mitigates Oxidative Damage Caused by Cd^2+^ Stress

**Figure 6:**
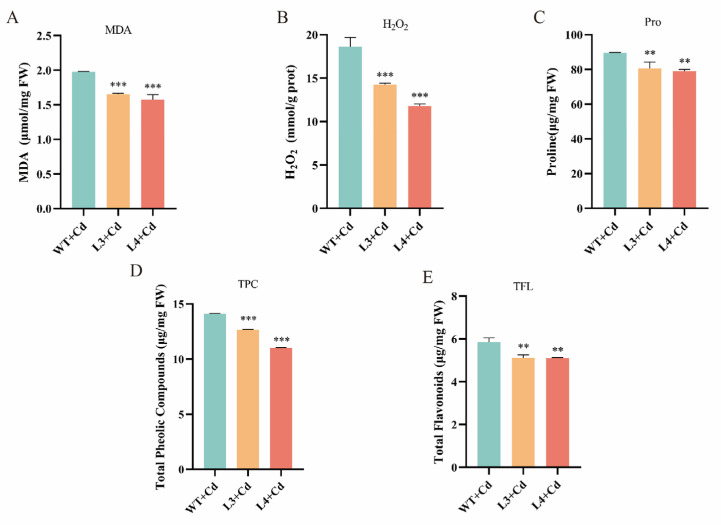
A comparative analysis of oxidative damage extent (MDA, H_2_O_2_, Pro), along with an assessment of non-enzymatic antioxidants levels (TPC and TFL), in transgenic and wild-type tobacco plants under cadmium stress. (**A**) MDA. (**B**) H_2_O_2_. (**C**) Pro. (**D**) TPC. (**E**) TFL. All statistical analyses were performed by one-way ANOVA, where ** represents *p* < 0.01, *** represents *p* < 0.001, *n* = 3.

To assess oxidative damage in tobacco under Cd^2+^ stress, this study measured the levels of MDA, H_2_O_2_, and proline. The transgenic tobacco exhibited significantly lower levels of MDA, H_2_O_2_, and proline compared to WT (Figure 6A–C). Specifically, in transgenic lines L3 and L4, the MDA levels were 83% and 77% of the wild type, H_2_O_2_ levels were 73% and 65% of the wild type, and proline content was 90% and 88% of the wild type, respectively. These results indicate that overexpression of NtGPX8a significantly alleviates oxidative damage caused by Cd^2+^ stress in tobacco. Elevated levels of ROS typically induce the accumulation of non-enzymatic antioxidants such as total phenolic content (TPC) and total flavonoid content (TFL). Consequently, we measured the TPC and TFL contents to reflect ROS levels. As anticipated, the levels of TPC and TFL in transgenic plants L3 and L4 were significantly lower than in the wild type. These findings collectively suggest that the overexpression of *NtGPX8a* effectively mitigates oxidative damage induced by Cd^2+^ stress in tobacco.

### 3.7. Impact of NtGPX8a Overexpression on Antioxidant Enzyme Activities

**Figure 7:**
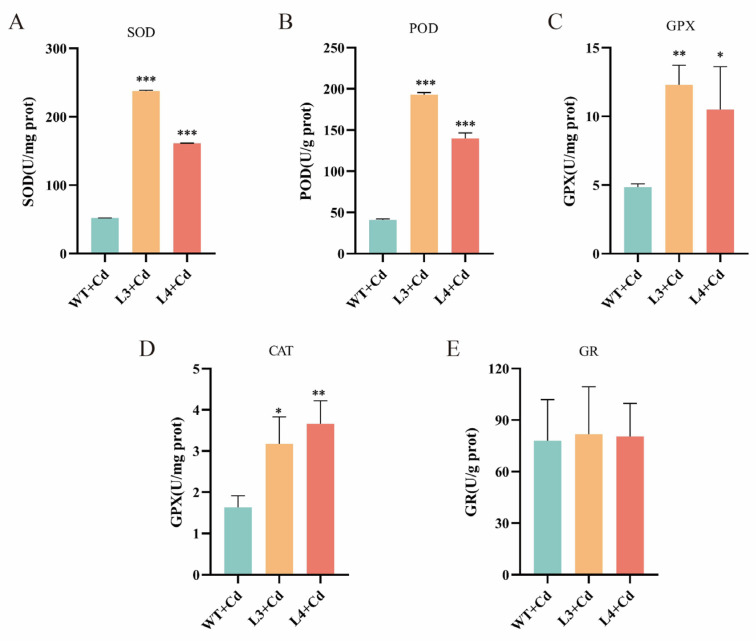
Determination of major antioxidant enzyme activities (GPX, SOD, CAT, POD, GR) in tobacco leaf discs under Cd^2+^ stress. (**A**) Total GPX enzyme activity. (**B**) SOD enzyme activity. (**C**) POD enzyme activity. (**D**) CAT enzyme activity. (**E**) GR enzyme activity. All statistical analyses were performed by one-way ANOVA, where * represents *p* < 0.05, ** represents *p* < 0.01, *** represents *p* < 0.001, *n* = 3.

The plant’s antioxidant enzyme system plays a pivotal role in eliminating Cd-induced ROS accumulation. To validate whether the overexpression of *NtGPX8a* triggers a synergistic effect in antioxidant enzymes, we measured the activities of GPX, SOD, CAT, POD, and GR in tobacco leaf discs under Cd^2+^ treatment. In transgenic tobacco lines L3 and L4, the SOD activities were 4.59- and 3.10-fold higher than the wild type (Figure 7A), while the POD activities were 4.71- and 3.49-fold higher, respectively (Figure 7B). The GPX activities were 2.41- and 2.13-fold higher, and CAT activities were 1.75- and 1.98-fold higher than the wild type (Figure 7C,D). However, the GR activity in transgenic tobacco did not show a significant difference compared to the wild type (Figure 7E). Overall, except for GR, the SOD, POD, GPX, and CAT activities in transgenic tobacco were significantly higher than in the wild type. The synergistic action of these antioxidant enzymes effectively explains how the overexpression of NtGPX8a alleviates Cd-induced oxidative stress in transgenic tobacco.

## 4. Discussion

### 4.1. NtGPX8a Is the Primary Member of the NtGPX Family in Response to Cd^2+^ Stress

The functional diversity among gene family members better meets the needs of plant growth, development, and adaptation to complex and changing environmental factors. The plant *GPX* gene family comprises a small gene family encoded by no more than 30 members. In this study, employing a whole-genome analysis strategy, we identified 14 *NtGPX* gene family members in the tobacco variety TN90 (Table S1). Subcellular localization analysis revealed that these genes are distributed among organelles such as the cytoplasm, chloroplasts, mitochondria, and nucleus, akin to the subcellular localization of *Arabidopsis* AtGPX [42]. Indeed, multiple organelles within plant cells generate reactive oxygen species (ROS) in response to various environmental stresses [43]. Hence, the localization of *NtGPX* family members within specific organelles aids tobacco in responding to ROS production induced by different environmental stressors.

The phylogenetic tree demonstrates the clustering of NtGPX members into five groups, where within the G1 group, three members of the NtGPX3 subfamily do not cluster with *Arabidopsis* AtGPX3 (Figure 1). Furthermore, multiple sequence alignment (Figure S1) and conserved domain analysis (Figure S2) reveal the absence of motif1 in NtGPX3 subfamily members, suggesting a potentially unique evolutionary trajectory for NtGPX3. Two members of the NtGPX8 subfamily cluster with PtGPX5 from poplar. Notably, the crystal structure of PtGPX5 indicates its capability to scavenge Cd-induced ROS and serve as a heavy metal sink by chelating Cd ions [28]. Additionally, studies indicate that the catalytic activity of PgGPx is not compromised by the presence of Cd in *P. glaucum* [29]. These findings suggest that certain members within the plant GPX protein family appear to have functions extending beyond the scavenging of ROS under stresses.

To elucidate the expression patterns of *NtGPX* family members under Cd^2+^ stress, qPCR analysis was conducted on tobacco *NtGPX* family members (Figure 2B). Initially, during the onset of Cd treatment, the expression levels of most *NtGPX* genes showed downregulation, akin to the expression pattern of *CcGPX* in *Cyprinus carpio* under Cd^2+^ stress [44]. As anticipated, the expression level of NtGPX8a was notably induced by Cd, compared to other *NtGPX* family genes. This finding is consistent with observations in *Caenorhabditis elegans* [45], suggesting a specific induction of *GPX8* subfamily member expression under Cd^2+^ stress. In summary, Cd^2+^ stress does not uniformly affect the expression patterns of all plant *GPX* gene family members, with *NtGPX8a* emerging as the predominant gene responsive to Cd^2+^ stress in tobacco.

### 4.2. NtGPX8a Selects Special Thioredoxins as the Electron Donor

Most animal GPX enzymes require the consumption of two GSH molecules as electron donors to reduce H_2_O_2_ [45]. In contrast, plant GPX relies on thioredoxins (Trx) as electron donors to alleviate oxidative stress induced by environmental pressures [46,47,48]. In fact, the utilization of Trx by plant GPX to alleviate oxidative stress is crucial for combating environmental adversities, given that extreme oxidative stress can lead to a decreased GSH/GSSG ratio and excessive GSH consumption [49]. A widespread and critical function of thioredoxins is their role as electron donors for peroxidases to reduce H_2_O_2_ [50]. Plants recruit specific thioredoxins to combat specific environmental stressors, indicating selective behavior of plant thioredoxins towards substrates [51]. Based on Zhang et al.’s findings [39], we selected three potential interactors of *Arabidopsis* Trx proteins (AtTrxZ, AtTrx5, and AtTrxm2) that might interact with NtGPX8a for yeast two-hybrid testing. The results showed that NtGPX8a can interact with AtTrxZ and AtTrxm2, but not with AtTrx5 (Figure 4C). This selectivity might depend on whether plant GPX and Trx exist in the same subcellular compartment [27]. Subcellular prediction in this study showed that NtGPX8a localizes in the cytosol or chloroplasts (Table S1). Interestingly, AtTrxm2 and AtTRxZ are predicted to localize in the chloroplasts [52], whereas AtTrx5 is a secretory protein present in the extracellular matrix that plays a crucial role in intercellular communication [53,54]. It is noteworthy that whether the selectivity of plant GPX for Trx depends on the assumption of subcellular co-localization requires further experimental confirmation.

### 4.3. Overexpression of NtGPX8a Alleviates Cd-Induced Oxidative Stress in Transgenic Tobacco

The visible indicator of plant damage under Cd^2+^ toxicity is typically chlorosis. In comparison to the WT, tobacco leaf discs overexpressing NtGPX8a showed a significant increase in photosynthetic pigment content under Cd stress (Figure 5C). This result suggests that the expression of NtGPX8a contributes to mitigating the deleterious effects of Cd^2+^ toxicity on photosynthetic pigments. Moreover, three physiological markers associated with oxidative stress, including MDA, H_2_O_2_, and proline, exhibited significantly reduced levels in the transgenic tobacco leaves (Figure 6). These findings further confirm that overexpression of *NtGPX8* can effectively alleviate Cd-induced oxidative stress. Plants typically utilize peroxidases or non-enzymatic substances, such as total phenols and total flavonoids, to scavenge H_2_O_2_ and other peroxides to maintain cellular ROS homeostasis [55].

Cd^2+^ stress significantly induces the accumulation of total phenols and total flavonoids in plants as a response to oxidative stress [56,57]. In transgenic tobacco overexpressing *NtGPX8*, the levels of total flavonoids and total phenols were significantly lower compared to the Cd-treated WT leaf disks (Figure 6D,E), possibly due to the alleviation of oxidative damage in tobacco caused by overexpressing *NtGPX8*, leading to reduced demand for total flavonoids and total phenols. Plants have evolved a comprehensive antioxidant enzyme system to cope with various environmental stresses. Cd’s regulation of antioxidant enzymes manifests in two aspects: firstly, by activating or inhibiting antioxidant enzymes gene expression through ROS signaling [21]; secondly, Cd^2+^ reduces the activity of antioxidant enzymes by displacing essential metal ions in the enzyme [58]. In this study, compared to the Cd-treated WT leaf disks, overexpressing *NtGPX8a* not only effectively increased NtGPX8a enzyme activity but also significantly enhanced the activities of SOD, POD, and CAT in transgenic tobacco (Figure 7). A plausible explanation is that approximately 90% of antioxidant enzymes activity in plants is attributed to GPX [59], and the overexpression of *NtGPX8a* efficiently alleviates Cd’s inhibitory effect on other antioxidant enzymes. Notably, GR enzyme activity was not affected by the overexpression of *NtGPX8a*, possibly because the NtGPX8a-dominated antioxidant system efficiently scavenged ROS induced by Cd^2+^, and the transgenic tobacco did not excessively deplete GSH produced from the ASA-GSH cycle, hence no significant change in GR enzyme activity occurred.

### 4.4. NtGPX8a Could Serve as a Candidate Gene for Engineering a High-Tolerance Cd Hyperaccumulator

Phytoremediation, recognized for its economic and environmentally friendly advantages, is considered a viable technique for remediating heavy metal-contaminated soil [14,60]. However, the toxicity of heavy metals to hyperaccumulators remains a crucial factor limiting the efficacy of phytoremediation [61]. Hence, the creation of a Cd-tolerant hyperaccumulator via genetic engineering might be pivotal in widely applying phytoremediation for soil heavy metal control. Extensive research has been conducted on the antioxidant enzyme system’s ability to mitigate Cd-induced ROS, clearing the Cd^2+^ toxicity [62,63]. Nonetheless, studies have shown that the activities of antioxidant enzymes responsible for ROS scavenging, such as SOD, CAT, GR, and APX, are significantly inhibited by high Cd^2+^ concentrations, with GPX being the sole exception [64]. This study demonstrated that the overexpression of *NtGPX8a* in *E. coli* (Figure 3B,D) and tobacco (Figure 5A) effectively alleviated Cd^2+^ toxicity. Surprisingly, overexpression of *NtGPX8a* significantly increased Cd accumulation in both *E. coli* and tobacco (Figure 3C and Figure 5B), suggesting the chelation of Cd ions by NtGPX8a. Furthermore, PtGPX5, clustering with NtGPX8a, possesses 36 Cd ion-chelating sites, rendering it a heavy metal sink [28]. Additionally, PgGPX isolated from *P. glaucum* is a Cd-dependent peroxidase [29]. These findings imply that NtGPX8a not only functions in ROS scavenging but also exhibits a dual function in Cd chelation. Therefore, the overexpression of *NtGPX8a* in plants may lead to the creation of hyperaccumulator plants capable of tolerating high Cd^2+^ concentrations, thereby providing valuable germplasm resources for efficient soil heavy metal remediation through phytoremediation.

## 5. Conclusions

Through comprehensive genome-wide analysis, this study successfully identified 14 *NtGPX* family genes from the tobacco variety TN90. Analysis using a phylogenetic tree revealed the subdivision of the *NtGPX* gene family into seven distinct subgroups, where genes within each subgroup exhibited analogous conserved domains and exon–intron patterns. Notably, aside from the NtGPX3 subgroup, the sequences of other *NtGPX* family genes demonstrated substantial consistency. Further analysis of promoter *cis*-acting elements suggested a wide regulatory potential for the expression of *NtGPX* family genes, indicating responsiveness to various plant hormones and stress cues. Noteworthy among these genes was *NtGPX8a*, distinctly responsive to Cd^2+^ stress, utilizing AtTrxZ and AtTrxm2 as electron donors, in contrast to AtTrx5. Heterologous expression of *NtGPX8a* in *E. coli* significantly mitigated the growth-inhibiting effects of Cd^2+^ toxicity. In tobacco, overexpression of *NtGPX8a* notably alleviated Cd-induced damage to photosynthetic pigments, reducing the contents of oxidative stress-related indicators, including MDA, H_2_O_2_, and proline, significantly compared to the WT. Moreover, overexpressed *NtGPX8a* in transgenic tobacco exhibited notably higher activities of plant antioxidant enzymes (GPX, SOD, POD, and CAT), reducing the reliance on non-enzymatic substances like total flavonoids and total phenols under Cd^2+^ stress, except for GR. Of particular note, the expression of *NtGPX8a* in both *E. coli* and tobacco significantly promoted intracellular Cd accumulation. These findings underscore the dual role of *NtGPX8a* in enhancing Cd tolerance and facilitating Cd accumulation in tobacco in response to Cd^2+^ stress.

## Data Availability

All data are contained within the article or Supplementary Materials.

## Supplementary Materials

The following supporting information can be downloaded at: https://www.mdpi.com/article/10.3390/genes15030366/s1, Figure S1: Sequence alignment of the *NtGPX* family; Figure S2: Analysis of conserved domains and gene structures of the *NtGPX* family; Figure S3: Analysis of *cis*-acting originals of the *NtGPX* family; Figure S4: Identification and expression level analysis of OE-*NtGPX8a* tobacco; Table S1: Putative *NtGPX* family overall features in tobacco; Table S2: *Cis*-acting elements of *NtGPX* gene promoter regions; Table S3: qPCR primers; Table S4: Construction of a prokaryotic expression vector and primer design for cross-vector detection.
