# Supplementary material for: Overexpression of NtGPX8a Improved Cadmium Accumulation and Tolerance in Tobacco (Nicotiana tabacum L.)
Source: Genes (Basel). 2024 Mar 15;15(3):366. doi: 10.3390/genes15030366 (PMC10970676; doi:10.3390/genes15030366)
Supplement: Supplementary file 1 [file genes-15-00366-s001.zip › genes-2878262-supplementary.pdf]

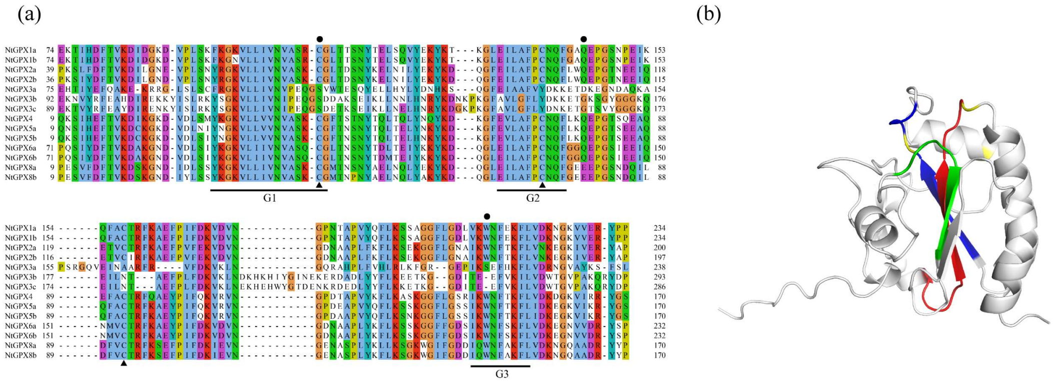

Figure S1. Multiple sequence alignment of the *NtGPX* gene family and 3D model of NtGPX8a. (a) The triangles denote three conserved Cys residues, while the circles represent the three amino acids Cys, Trp, and Gln that constitute the catalytic triad. G1, G2, and G3 indicate highly conserved characteristic domains. (b) 3D model of NtGPX8a. Red represents the G1 domain, blue represents the G2 domain, and green represents the G3 domain, Yellow represents critical Cys residues in NtGPX8a.

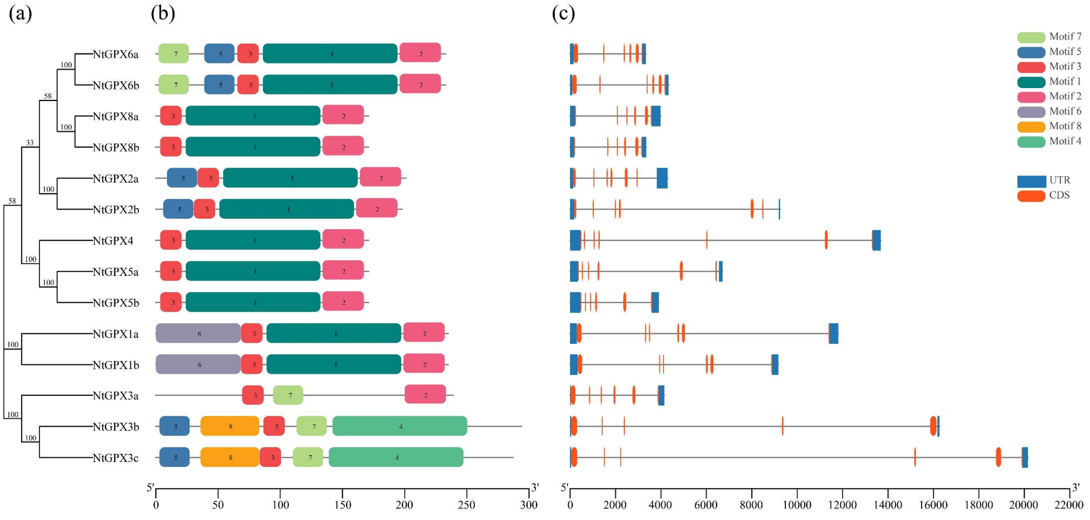

Figure S2. *NtGPX* family genes system evolutionary tree, conserved domains, and exon-intron structure: (a) Evolutionary tree of the *NtGPX* family system constructed using the neighbor-joining method; (b) Conserved domains of NtGPX proteins; (c) Gene structure of the *NtGPX* family, with introns represented by black lines, and UTR and CDS indicated by different colored boxes.

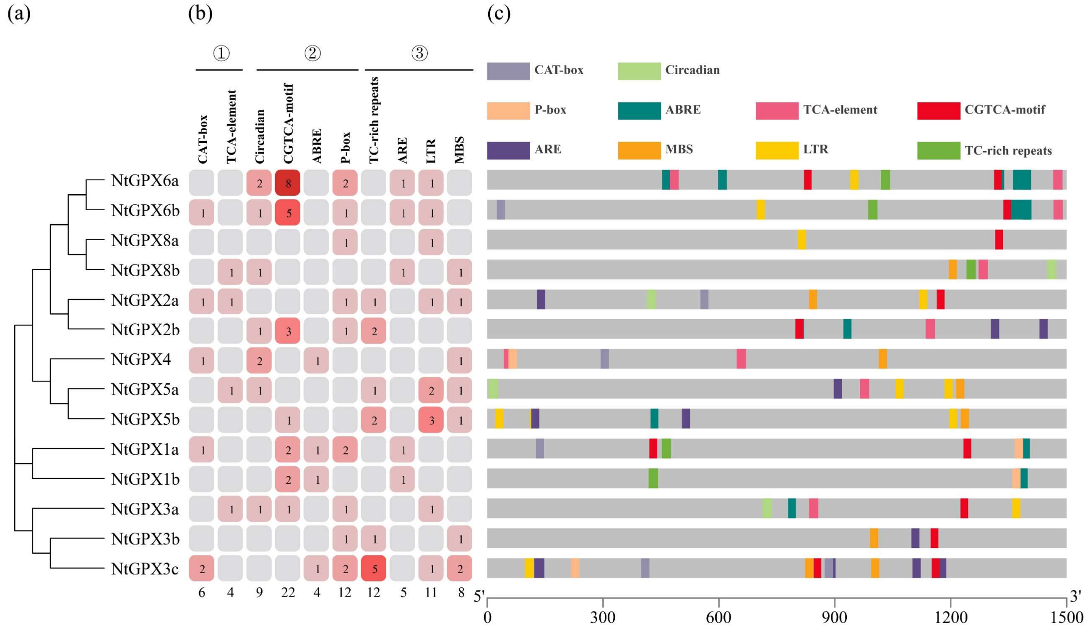

Figure S3. *NtGPX* family genes promoter *cis*-acting element analysis. The frequency of occurrence of *cis*-acting elements is represented by numbers and different colored boxes.

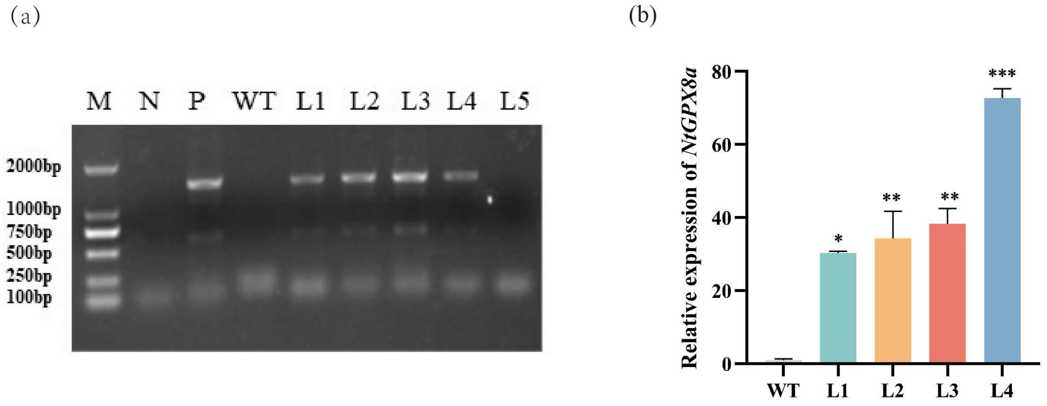

Figure S4. Identification of tobacco positive plants overexpressing *NtGPX8a*. (a) Identification of transgenic tobacco lines overexpressing *NtGPX8a*. (a) Genomic PCR electrophoresis of transgenic tobacco. M: 2000-bp DNA marker; N: negative control with sterile water as the PCR template; P: positive control with pCXSN::*NtGPX8a* plasmid as the template; the positive control lanes show two bands at 1460-bp and 606-bp, respectively. (b) Relative expression levels of *NtGPX8a* in different strains of tobacco (stars in the figure indicate statistically significant differences calculated using One-way ANOVA, * represents $p < 0.05$, ** represents $p < 0.01$, *** represents $p < 0.001$, n = 3).

Supplementary Table S1. Putative *NtGPX* family overall features in tobacco

Gene name	Gene ID	mRNA ID	CDS size (bp)	Amino acid size (aa)	MW(kDa)	pI	Subcellular localization prediction
NtGPX1a	gene_46900	mRNA_100233	708	235	25.79	9.36	Chloroplast. Mitochondrion.
NtGPX1b	gene_83324	mRNA_178811	708	235	25.73	9.39	Chloroplast. Mitochondrion.
NtGPX2a	gene_33363	mRNA_71469	606	201	22.83	7.63	Chloroplast. Mitochondrion.
NtGPX2b	gene_45308	mRNA_96996	597	198	22.45	6.37	Chloroplast. Mitochondrion.
NtGPX3a	gene_32960	mRNA_70560	720	239	27.03	8.94	Nucleus.
NtGPX3b	gene_46901	mRNA_100234	885	294	33.17	9.36	Mitochondrion.
NtGPX3c	gene_83263	mRNA_178677	864	287	32.36	9.04	Mitochondrion.
NtGPX4	gene_52083	mRNA_111240	516	171	19.10	9.5	Chloroplast. Nucleus.
NtGPX5a	gene_4916	mRNA_10500	516	171	18.96	8.79	Chloroplast. Nucleus.
NtGPX5b	gene_12872	mRNA_27583	516	171	18.89	9.08	Chloroplast.
NtGPX6a	gene_62683	mRNA_133920	702	233	26.00	8.96	Chloroplast.
NtGPX6b	gene_66346	mRNA_141800	702	233	25.97	9.01	Mitochondrion.
NtGPX8a	gene_14893	mRNA_31782	516	171	19.27	4.91	Cytoplasm.Chloroplast.
NtGPX8b	gene_84095	mRNA_180432	516	171	19.29	4.98	Cytoplasm.Chloroplast.

Supplementary Table S2. ***Cis*****-acting elements of** ***NtGPX*** **genes promoter regions**

**Element**	**Core sequence**	**Function annotation**	**No. of genes containing element**
TCA-element	TCAGAAGAGG	*cis*-acting element involved in salicylic acid responsiveness	9
ABRE	ACGTG	*cis*-acting element involved in the abscisic acid responsiveness	22
P-box	CCTTTTG	gibberellin-responsive element	4
CGTCA-motif	CGTCA	*cis*-acting regulatory element involved in the MeJA-responsiveness	12
ARE	AAACCA	*cis*-acting regulatory element essential for the anaerobic induction	12
TC-rich repeats	GTTTTCTTAC	*cis*-acting element involved in defense and stress responsiveness	5
LTR	CCGAAA	*cis*-acting element involved in low-temperature responsiveness	11
MBS	CAACTG	MYB binding site involved in drought-inducibility	8
CAT-box	GCCACT	*cis*-acting regulatory element related to meristem expression	6
Circadian	CAAAGATATC	*cis*-acting regulatory element involved in circadian control	4

Supplementary Table S3. qPCR primers

gene id	name	prime（5’ - 3’）
NtEF1α	qF-NtEF1α	TGAGATGCACCACGAAGCTC
	qR-NtEF1α	CCAACATTGTCACCAGGAAGTG
NtGPX1a	qF-NtGPX1a	GTCAATGTTGCTTCCAGATG
	qR-NtGPX1a	GATTAGATCCTGGCTCTTGAG
NtGPX1b	qF-NtGPX1b	AGGACTTGAGATTCTTGCTT
	qR-NtGPX1b	GTGCTGTGTTAGGACCAT
NtGPX2a	qF-NtGPX2a	CTTGTTCTTGCCTTGTTCTT
	qR-NtGPX2a	CCAGTATATCCTTGACAGTGA
NtGPX2b	qF-NtGPX2b	CTTGTTCTTGCCTTGTTCTT
	qR-NtGPX2b	CCCAATATATCCTTGACAGTG
NtGPX3a	qF-NtGPX3a	GGACAGAATCGCAGTATGA
	qR-NtGPX3a	CTAGCAGCATTAATCTCTACC
NtGPX3b	qF-NtGPX3b	CTGAGCAAGGTTCTGATGA
	qR-NtGPX3b	CCTGATTTGCCTGTTTCTTT
NtGPX3c	qF-NtGPX3c	CAGGCACATCAGTATATGGA
	qR-NtGPX3c	TCCTCTGTAATATCTCCTCCT
NtGPX4	qF-NtGPX4	AGGTTCCAGGATTAAGTGGAAT
	qR-NtGPX4	ATCGTAAGTTTCATAGACTGCAC
NtGPX5a	qF-NtGPX5a	TCGTCAATGTTGCTTCCA
	qR-NtGPX5a	TACCAGGCTCTTGCTTCA
NtGPX5b	qF-NtGPX5b	TGACTGAACTCCACAACAA
	qR-NtGPX5b	ACCATTAACTCGCACCTT
NtGPX6a	qF-NtGPX6a	CCAGCAAGCCTCAATCTAT
	qR-NtGPX6a	GTATATCTCGGTCAAGTCAGT
NtGPX6b	qF-NtGPX6b	ACTCCTCCGATTCCAACA
	qR-NtGPX6b	TTAGCATCCTTGACAGTGAA
NtGPX8a	qF-NtGPX8a	TCAGAGGAGAGGTGGTAGGAT
	qR-NtGPX8a	AGGGAGCAATGATCAGATCTTAGA
NtGPX8b	qF-NtGPX8b	TACGCAGAACTCAACCAAT
	qR-NtGPX8b	CACCATTCACTTCAATCTTG

Supplementary Table S4. Construction of a prokaryotic expression vector and primer design for cross-vector detection.

name	prime（5’ - 3’）
sF-T7 promoter	TAATACGACTCACTATAGGG
sR-pET28a	AGTTATTGCTCAGCGGTGGCA
F-NtGPX4-*BamH* I	cgGGATCCATGGGTGCTTCTAAATC
R-NtGPX4-*Hind* III	ccAAGCTTTCAATTCTCGCTTATTGCC
F-NtGPX5a-*BamH* I	cgGGATCCATGGGTGCCTCTTCAT
R-NtGPX5a-*Hind* III	ccAAGCTTTCACACTTCCCCTAG
F-NtGPX6a-*BamH* I	cgGGATCCATGCTTTGTTCTGTAAC
R-GPX6a-*Hind* III	ccAAGCTTTTAAGCAACACCCA
F-NtGPX8a-*BamH* I	ccAAGCTTCACGATTTCCAGCAGC
R-NtGPX8a-*Hind* III	cgGGATCCATGGCCAGCCAATCAGAGA
